# Supplementary figures and images for: Self-organization of a doubly asynchronous irregular network state for spikes and bursts
Source: PLoS Comput Biol. 2021 Nov 8;17(11):e1009478. doi: 10.1371/journal.pcbi.1009478 (PMC8575278; doi:10.1371/journal.pcbi.1009478)

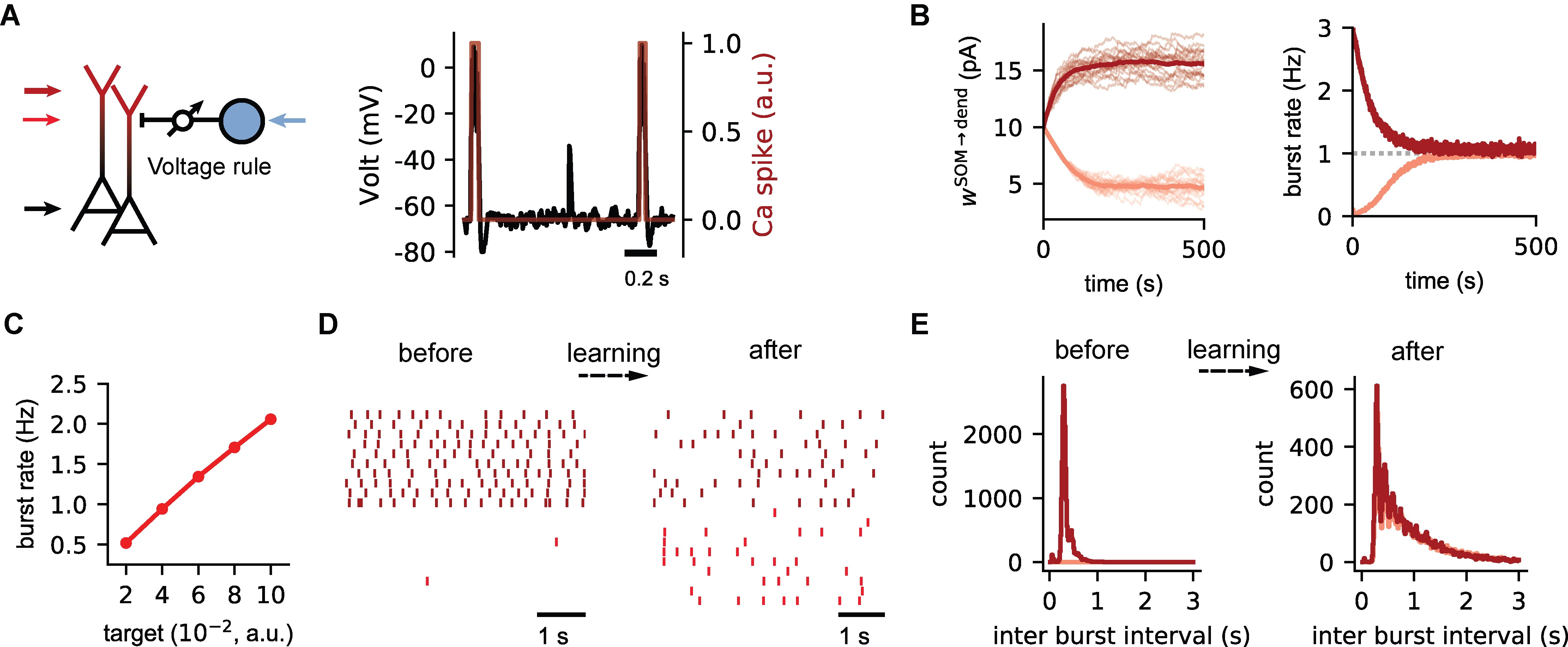

Supplement: S1 Fig — (A) Network configuration with distal dendrites of PCs under control of inhibitory synaptic inputs from SOMs (blue circle). The inhibitory connections are plastic (arrow) and modified according to a homeostatic plasticity rule where post-synaptic activity is modelled by a filtered version of the dendritic voltage (right, red trace)(Methods). (B) Bursts are activated by weak (light red, Iid250pA) or strong (dark red, Iid650pA) dendritic input with moderate noise levels (σd = 100 pA). The somatic input is the same for both dendritic inputs (Iis=500pA, σs = 100 pA). The target value was determined empirically (see C) so that the burst rate was 1 Hz (dashed line). (C) The burst rate after learning the inhibitory weights for different target values. (D) Representative raster plots of the burst activity for weak (light red) and strong (dark red) dendritic inputs, before and after learning. Each dot represents a burst. (E) The distribution of the inter-burst intervals (IBI) before and after learning for weak (light red) and strong (dark red) dendritic inputs. (TIF) [file pcbi.1009478.s001.tif]

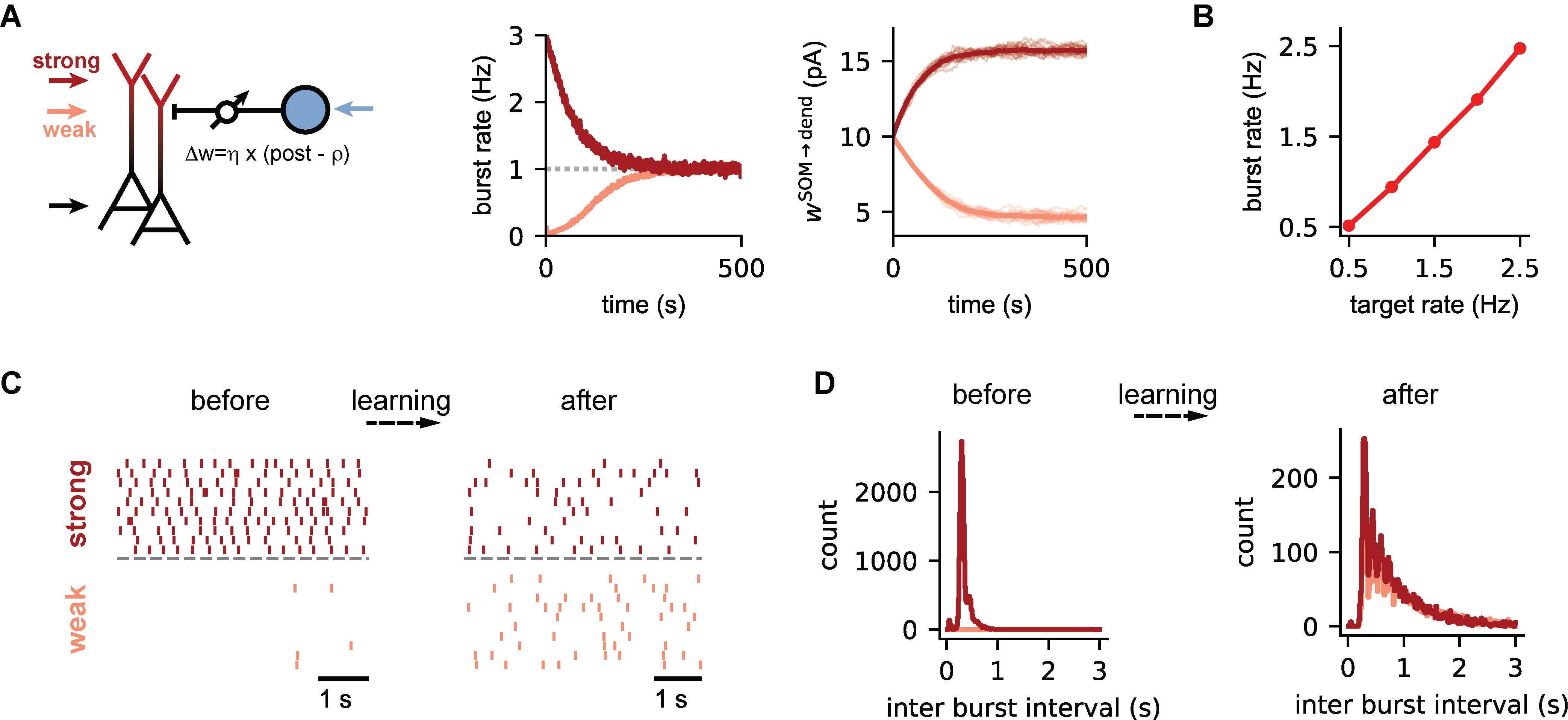

Supplement: S2 Fig — (A) Network configuration with distal dendrites of PCs under control of inhibitory synaptic inputs from SOMs (blue circle). Bursts are activated by weak (light red, Iid=250pA) or strong (dark red, Iid=650pA) dendritic input with moderate noise levels (σd = 100 pA). The somatic input is the same for both dendritic inputs (Iis=pA, σs = 100 pA). The strength of the inhibitory connections WSOM→dend are plastic (arrow) and modified according to a homeostatic plasticity rule dependent on dendritic post-synaptic activity (Methods). The burst target rate (dashed line) was set to 1 Hz. (B) The burst rate after learning the inhibitory weights for different target burst rates. (C) Representative raster plots of the burst activity for weak (light red) and strong (dark red) dendritic inputs, before and after learning. Each dot represents a burst. (D) The distribution of the inter-burst intervals (IBI) before and after learning for weak (light red) and strong (dark red) dendritic inputs. (TIF) [file pcbi.1009478.s002.tif]

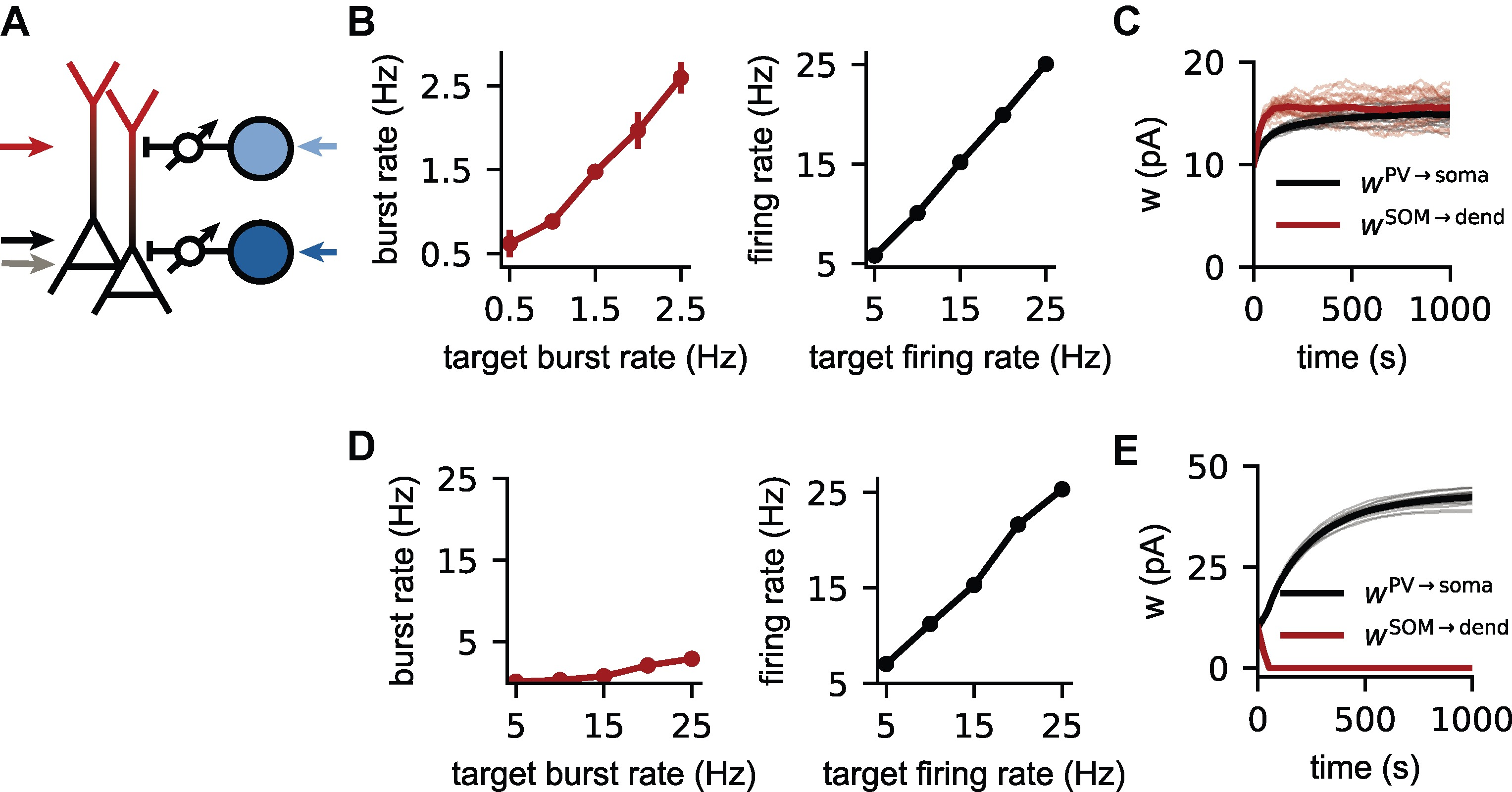

Supplement: S3 Fig — The somatic and dendritic activity of PCs is under control of plastic inhibitory connections from PV (dark blue) and SOM (light blue) interneuron populations (see Fig 2). The somatic and dendritic compartments receive strong external inputs with moderate noisy background input. (Iid=650pA, Iis=1100pA, σd = σs = 100 pA). (B, C) No competition (target firing rate = 10 times target burst rate) versus (D,E) competition (target firing rate = target burst rate) between the target burst rate and target firing rate. (B,D) The burst and firing rate for different burst and firing target rates after learning the inhibitory weights (C,E). (TIF) [file pcbi.1009478.s003.tif]

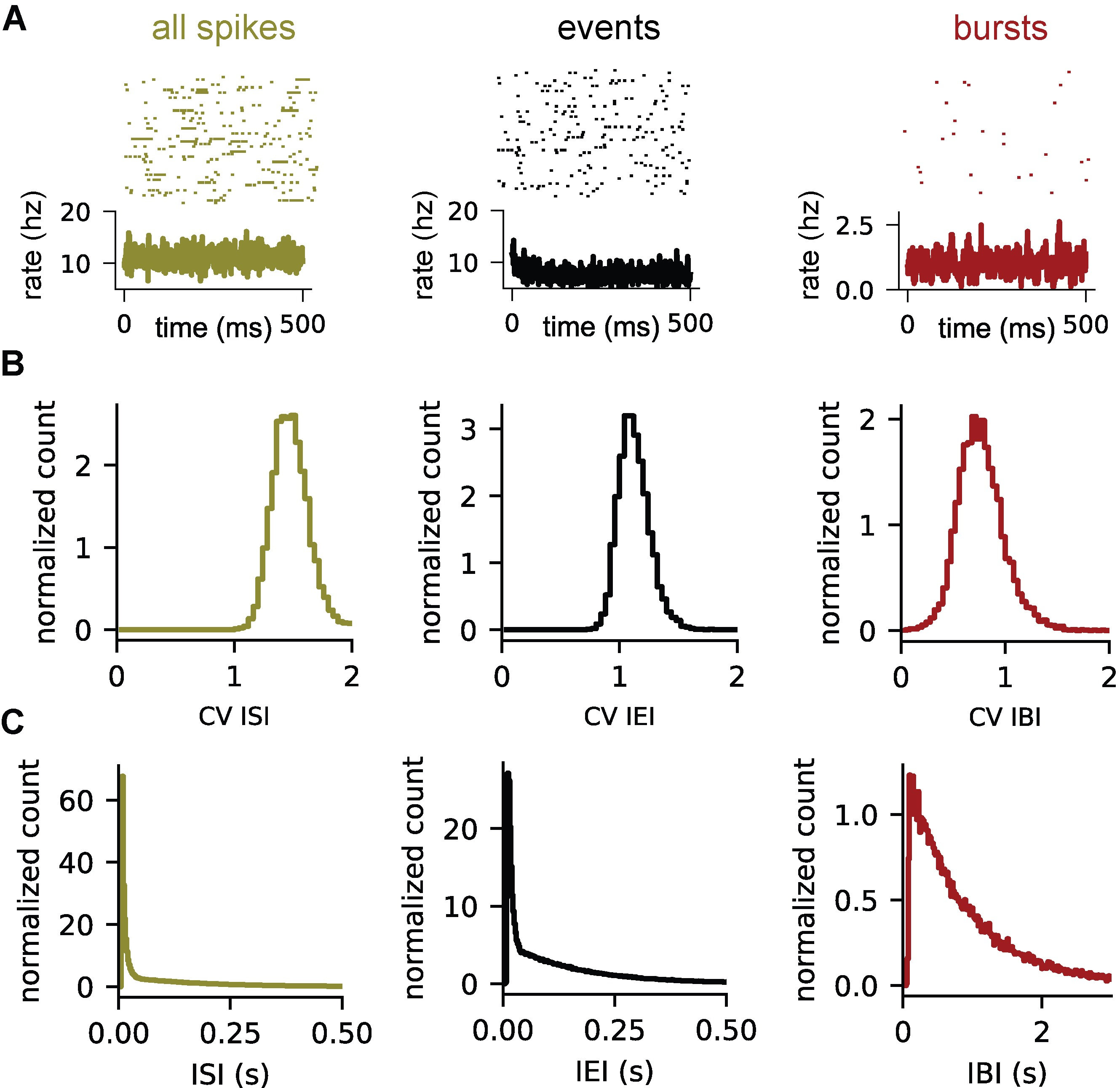

Supplement: S4 Fig — The network configuration and stimulus condition are the same as for Fig 3 (A) (Top) Representative raster plots of all spikes, events and bursts of 50 neurons after learning the inhibitory weights. (Bottom) Histogram of all spikes, events or bursts of the entire PC population, normalized by the number of neurons (8000) and binsize (1 ms) to have units of rate. (B) The distribution of the coefficient of variation of the inter-spike intervals (CV ISI, yellow), inter-event intervals (CV IEI) and inter-burst intervals (CV IBI) after learning the inhibitory weights. (C) The distribution of the inter-spike intervals (ISI, yellow), inter-event intervals (IEI) and inter-burst intervals (IBI) after learning the inhibitory weights. (TIF) [file pcbi.1009478.s004.tif]

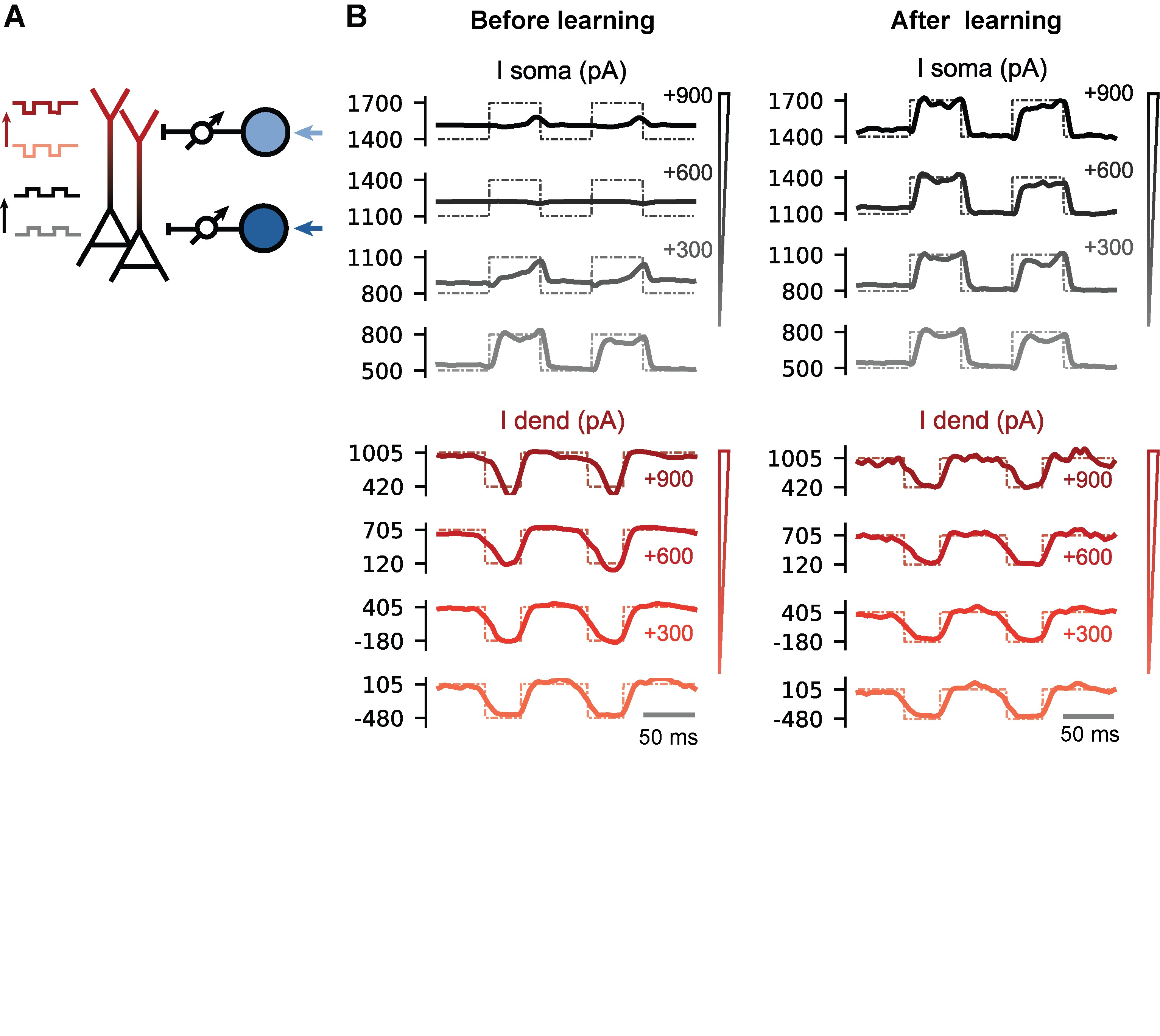

Supplement: S5 Fig — (A) Stimulation paradigm with an increase in background excitation (triangle, red = dendrite, black = soma) on which pulse inputs are superimposed. Similar to Fig 4, alternating and opposite pulse inputs (dashed lines) are delivered to the somatic and dendritic compartment (Iis,high=800pA, Iis,low=500pA, Iid,high=105pA, Iid,low=−480pA, σis=σid=450pA) and the dendritic and somatic background is increased by 300, 600 and 900 pA, respectively. Plastic inhibitory connections from PV (dark blue) and SOM (light blue) interneuron populations restore the multiplexed burst code without the need for fine-tuning the background input. (B) Decoded input currents from the event rate (solid red) and burst probability (solid black) before and after learning (see Methods). Dashed lines represent the actual dendritic and somatic inputs. (TIF) [file pcbi.1009478.s005.tif]
