# Supplementary material for: Self-organization of a doubly asynchronous irregular network state for spikes and bursts
Source: PLoS Comput Biol. 2021 Nov 8;17(11):e1009478. doi: 10.1371/journal.pcbi.1009478 (PMC8575278; doi:10.1371/journal.pcbi.1009478)
Supplement: Attachment — Submitted filename: Responses to Reviewers.pdf [file pcbi.1009478.s006.pdf]

Response to reviewer comments
(Vercruysse & Sprekeler, "Self-organization of a doubly asynchronous irregular network state for spikes and bursts")

We would like to thank the reviewers for the thorough read and the constructive comments, which helped us a lot to improve the paper. Please find below the detailed responses to the comments of the reviewers.

Reviewer 1

Major concern:

My major concern is that some parameters of the model that change between results are not sufficiently justified: For example, and of relevance to the results described in lines 224-239, the learning rate of SOM->d is higher than the learning rate of PV->s in Fig. 2,4 and lower in Fig. 3. An intuition/description of the different behaviors when using the respective values will increase their understanding.

Another such example is in Fig. 3 (and Fig. 4), whereby the authors chose not to excite SOM with external inputs. What is the reasoning for this, or else, when/why does the feedforward excitation of the SOM interneurons prohibit the generation of asynchronous states/multiplex code?

Thank you, that's an important point. We now comment more extensively on the parameter choices in the methods section. Regarding the learning rates, we now run the simulations in Fig. 3 with the same learning rates used in Fig. 4, to show that the results are robust. We also added the following paragraphs to the methods section (l. 599):

> *The learning rates were varied for several reasons. First, in Fig. 2, we chose the learning rates such that the two forms of plasticity occur on similar time scales, for illustration purposes. Second, some of the simulations are computationally costly, because they entail the simulation of spiking networks over the long time scales required for plasticity. We therefore maximised the learning rate for all conditions in which we performed extensive parameter sweeps, to reduce the required simulated time. Third, while the*

steady state should in principle be independent of the learning rate, this is not necessarily true in practise. For example, the recurrent networks in Fig. 3 are (deliberately) initialised in a high-activity, highly synchronous state, in which the notion of bursts is useless. To make sure that this state does not generate pathological weight distributions, we used a high learning rate for the soma-targeting inhibitory synapses, such that the overall activity of the network is rapidly reduced.

Regarding the choice of the background inputs, for Fig 3 and 4 we now also added the same background input to the SOMs as we did to the PVs to show that the results are robust. We also added the following paragraph to the Methods (l. 624):

A key point of the recurrent network simulations in Fig. 3 & 4G was to show that the network can maintain an AI state based on "internally generated noise" and thereby support a multiplexed code. We therefore removed all external noise (\sigma^{s, d, SOM, PV}=0). The amount of external input to the PV interneurons should not be chosen too low, because it indirectly controls the amount of recurrent inhibition among the PV interneurons: Because homeostatic plasticity on the recurrent inhibitory connections among the PV interneurons aims to achieve a given target rate, higher input leads to stronger inhibitory recurrence. If the overall excitatory input is too small, recurrent inhibition is removed altogether, which in turn increases the risk of PV interneurons to synchronise and thereby generate network oscillations.

The amount of external input to the SOM interneurons is less critical for the behaviour of the network as long as the relative input of recurrent inputs from the PCs is high enough. In contrast to the PV interneurons, SOM interneurons cannot use recurrent inhibitory connections to desynchronise their activity. We omitted those connections because they are rare in cortex (e.g., Pfeffer et al., 2013). A desynchronization of the SOM interneurons can therefore only be reached by input fluctuations. Because only the internal recurrent excitation fluctuates, but not the external inputs, the recurrence must account for a sufficiently large fraction of the SOM input to prevent network oscillations. Note that this is a somewhat artificial constraint. In the cortex, there would be both noise from the surrounding network activity and inhibition from other interneurons (e.g., VIP neurons; Pfeffer et al., 2013).

Minor:

Line 127-129: The authors state that: “we expected that the dynamic range of burst activity is limited when bursts are triggered by excitatory synaptic potentials on the apical dendrite alone”. Do they authors mean burst generation only due to calcium channels in the apical dendrites (without the coincidence of somatic AP), or bursts that do not dependent on e.g. persistent sodium channels at the soma?

Yes, that sentence was not phrased very clearly. We hope that the new version is more precise (l. 132):

> *Due to the non-linear nature of the burst generation mechanism, we expected that the dynamic range of burst activity is limited when bursts are driven by dendritic excitation alone (Softky & Koch, 1993): The difference between no burst activity and a situation where every somatic spike is dendritically amplified to a burst (Larkum et al., 1999) should be brought about by small differences in dendritic input.*

The pyramidal cell model was originally designed to describe the nonlinear interaction of somatic and dendritic inputs, but you're of course correct that for sufficiently strong dendritic inputs, it can generate bursts without somatic input. While that would change the way dendritic inputs are encoded in the postsynaptic spike train (cf. Fig. 4, but that's beyond the present paper), it does not prevent the suggested dendritic plasticity from controlling burst activity:

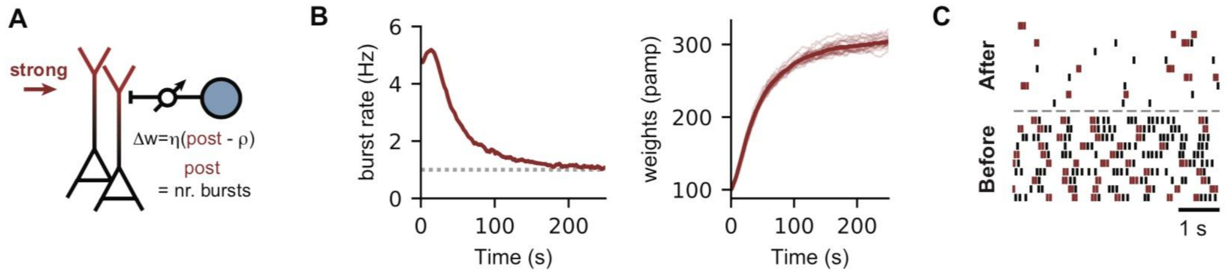

Line 217: For ease of reading, please indicate the difference of the two rules (difference in target rates, difference in learning rates).

We agree that providing additional detail on the key difference (namely the nature of the targets) between the two rules will increase the readability of that section. Therefore we changed the text to (l. 221):

> *To control both somatic and dendritic activity, we have distinct rules for the two inhibitory connections. SOM→dendrite connections are subject to the plasticity rule in Eq. 1, while a different spike timing-dependent inhibitory plasticity rule (Vogels et al., 2011) in the PV→somatic connection controls the overall level of activity (Fig 2A). The two learning rules have separate target rates (e.g., a burst rate of 1 Hz and an overall firing rate of 10Hz) and different learning rates (see Methods for further details).*

Fig. S3, panel labeling seems wrong.

We thank the reviewer for pointing out this mistake. We have corrected the labels.

Line 474: Please correct to nS (instead of pA).

Thanks, this should be correct now.

Fig. S4 is not referenced in the main text.

Thanks, we added the reference in l. 268:

> *To assess the degree of irregularity of the activity without confounds from the presence of bursts, we studied the statistics not of individual spikes, but of events (Naud and Sprekeler, 2018, see Fig S4 B for a comparison of inter-spike interval and inter-event interval statistics).*

Line 600: Please delete parenthesis.

Thanks, corrected.

Reviewer #2: The authors propose a neural network model with two inhibitory learning rules to achieve a target number of bursts and overall activity. Based on previous inhibitory learning rules they propose a new one based on establishing a target burst rate, performed by SOM-like interneurons. This is then combined with a PV-like interneuron to balance out the overall activity with a standard ISP learning rule. The authors then show that this combination is effective at developing an asynchronous state with a combination of single spikes and bursts and seems to address some of the problems with current multiplexing models. This is an interesting study that adds important contributions to our knowledge of multiplexing and bursting more generally in cortical microcircuits. However, it falls short in some aspects that we highlight below, in

particular a better explanation of some elements (see below) and a lack of comparison with burst statistics observed experimentally.

Major points

1. For a general audience it would be good to clarify what is meant by a "threshold-like process" (e.g. abstract and intro), as this is central to the argument. At least in the introduction it would be good to expand on this so as to make it clear what encodes this threshold. This term seems later to be changed to non-linear, it would be good to link them or keep them consistent throughout the paper.

That's a very good suggestion. We extended the introduction as follows (l. 68):

> *For bursts to convey information effectively, they need to occur rarely, but consistently (Naud and Sprekeler, 2018). Neural recordings suggest that this is indeed the case (De Kock and Sakmann, 2008; Takahashi et al., 2016; Doron et al., 2020). However, such a condition is not easily established (Softky & Koch, 1993), because bursts are often triggered by nonlinear, threshold-like processes. For example, in PCs, bursts can be generated by dendritic calcium spikes, which in turn arise from the activation of voltage-gated calcium channels (London & Häusser, 2005). These channels activate a positive feedback loop upon sufficient dendritic depolarization, effectively introducing a threshold-like condition for the generation of bursts. If dendritic input is too low, bursts will be absent entirely. If it is too high, bursts will be the predominant form of activity. ...*

2. It is not clear from the results in Fig. 4 whether this effect is specific to *only* one of the event rates. I.e. does the model after learning have a reduced multiplexing error across input rates or is this only specific to one of the them as shown in G, and there is actually a reduction for the other input values (as in D)? This should be clarified.

Thanks, very well spotted. We now quantify the quality of the code for the two signals separately in Fig. 4I, in terms of the development of two correlation coefficients over time. For the fully recurrent case (Fig. 4I), we see a rapid improvement in both signals.

Why not show the model output for all the cases in D after learning?

Excellent suggestion, that underlines the robustness of the learning effect. To not overload Figure 4, we have now added a supplementary figure S5, in which the effect of learning for the other input levels is shown. Your comment also showed us that the description of Fig. 4 in the text is too brief, because we did not emphasise enough that the learning case in panels F-I is done in a recurrent setting and without external noise, whereas panels C-E are done for the feedforward setting with external noise studied by Naud et al. (2018). We updated the main text as follows (l. 310):

Given that the two forms of plasticity tend to establish a fluctuation-driven regime, we hypothesised that they could provide a basis for a self-organization of a multiplexed event-burst code. We first tested in an uncoupled PC population if somatic and dendritic homeostatic plasticity can compensate for the input level-dependent disruption of the multiplexed code. Indeed, the code is recovered over the course of learning for a broad range of input levels (Fig S5). Finally, we checked if the code can also be enabled in a fully recurrent setting without external noise (cf. Fig 3). Again, we find that inhibitory plasticity can compensate for inappropriate baseline currents (Fig 4F-I). Over the course of learning, the plasticity recovers a fluctuation-driven state(Fig 4G), and the resulting irregular asynchronous activity enables the multiplexed code (Fig 4H,I).

In addition, in Fig 4E and 4I why is multiplexing accuracy measured this way? Wouldn't it be better to use some form of decoder accuracy?

Thanks, that would indeed be more appropriate. Instead of reporting the overall error, we now report the correlation coefficient between the two input currents and the event rate/burst probability, respectively (Fig 4E,I). That has the advantage over errors that it puts the two quality measures on the same scale. To provide a visual comparison of the signals, we performed a linear regression (basically, a linear decoder) to rescale and shift event rate and burst probability to best match the two input currents (Fig. 4 B,D,H).

3. In the abstract it is stated "thereby increasing the information encoded in bursts". This appears to never be quantified. The authors could in principle use their previous measure to quantify this. Having this as a new figure would strengthen their point.

We appreciate that it would in principle be great to show the development of mutual information between input and output over learning. Unfortunately, this is not trivial for spiking data. Even in our earlier work (Naud et al., 2018), we shied away from that and merely plotted analytical results for mutual information, which can be readily obtained under simplifying assumptions on the coding properties of the PCs. We would kindly ask the reviewers to save us the effort, because we feel that the cost-benefit trade-off is not great.

4. A potential problem with this network with bursting is that runaway activity could easily develop before balance is established. This is particularly problematic as ISP learning rules typically need a low learning rate. A solution to this problem has recently been put forward based on developmental STP (Jia et al. BioRxiv 2021). This issue should be discussed.

In fact, the runaway activity you refer to is the initial condition in Fig 3, and is gradually abolished by the two forms of plasticity, just as in earlier work on inhibitory synaptic plasticity (e.g., Vogels et al., 2011). We appreciate that nature will likely have developed additional faster control loops that stabilize the network on a much faster time scale, be it by short-term depression as in Jia et al. (2020) or by other mechanisms. We now include a paragraph in the discussion (l. 425):

While inhibitory plasticity is a promising candidate for network homeostasis, it operates on a slow time scale of hours or days and is therefore likely complemented by additional negative feedback loops that operate on faster time scales (Zenke and Gerstner, 2017). Potential mechanisms that could rapidly stop network instabilities in their tracks could be, e.g., short-term synaptic plasticity (Anwar et al., 2017, Jia et al., 2021) or presynaptic inhibition (Naumann & Sprekeler, 2020). An inclusion of these mechanisms would be interesting, but exceeds the scope of the present study.

5. One of the motivations stated for this study (e.g. Intro) is to get closer to in vivo like statistics of bursts, but I failed to see a comparison or at least a detailed discussion on this point.

That's a very good point, thanks for pointing it out. We now added the following paragraph to the discussion (l. 432):

Relation to in vivo bursting statistics. *In vivo, bursts occur rarely, but consistently. In rat somatosensory cortex, the proportion of spikes that occur in bursts is about 15-20% (De Kock et al., 2008). Similar values were reported by (Doron et al., 2020). Sanders et al. (2019) found a slightly higher, but quite variable proportion of about 40% in rat CA1 and CA3. While firing and burst rates vary substantially across brain regions, the proportion of bursts hence seems to be similar across brain regions. Note that a direct quantitative comparison of these numbers is problematic, because not all papers use the same formal criteria for the classification of bursts. In our simulations, we chose the target rates of the plasticity rules such that the proportion of spikes occurring in bursts roughly aligns with the experimental observations. For a firing rate of 10Hz, a burst rate of 1Hz and 2-3 spikes per bursts, about 20-30% of the spikes are part of a burst.*

6. What are the implications for biological plausibility of removing the somatic adaptation to allow for shorter ISI in Fig 3? More importantly, why include the somatic adaptation in the other figures if it is removed in fig 3?

We agree that this model choice appears somewhat arbitrary. We now discuss this point in detail in the methods, hoping that this justifies the model choice well enough (l. 514):

The choice to remove adaptation for Fig 3 is a classical trade-off between clarity of result and biological accuracy. The original PC model of Naud et al. (2014) contains somatic adaptation because this allows a closer fit to neural recordings. Therefore, the biologically more accurate model choice should probably include adaptation. On the other hand, adaptation suppresses short ISIs and thereby generates more regular firing (Gerstner et al., 2014). As a result, the CV of the inter-event distribution would be consistently below the value 1 that is considered the hallmark of irregular activity. Unfortunately, this could readily be mistaken for an indication that the network is not a fluctuation-driven state. Therefore, we removed the adaptation to make the result on the double AI state as clean as possible. Putting adaptation back in does not drastically

change the network state, but the CV of the inter-event distribution is consistently below 1.

Minor points

1. Fig 3B: You state that “Strong constant somatic (Is i = 1500 pA) and dendritic (Id i = 500 pA) external input drives L5 PCs to fire synchronously at high rates without burst activity” but the plot shows firing that has an ISI below 16ms (~4ms) which suggests that it’s all bursting activity. Maybe this is just a question of rewording?

Yes, that's a good point. We changed the caption as follows:

Strong constant somatic (Is i = 1500 pA) and dendritic (Id i = 500 pA) external input drives L5 PCs to fire synchronously at high rates. No clearly separated bursts are detectable.

and corrected our definition of burst activity in the Methods section (Data Analysis subsection, l. 647):

Bursts are defined as a set of spikes where the interspike interval (ISI) is smaller than 16 ms, followed by a period of quiescence before the next burst occurs.

2. Line 135: distal dendritic compartment?

Yes, it's probably a good idea to make this explicit, but we decided to do so even earlier at the beginning of the Results section, where the PC model is described (l. 111):

In short, the PC model contains a somatic compartment and a dendritic compartment that describes the distal apical dendrite. Both are each modelled by a set of differential equations [...]

3. Missing arrow (or some other symbol) onto the soma of the SOM and PV interneurons (Fig. 1 and 2) to indicate the background input.

Thanks. We added arrows to all neural populations that receive external input, in all figures.

4. There are other interneurons that target distal dendrites, namely the NDNF interneurons. Is there a reason for assuming that the ones modelled here are SOM?

We previously hinted at these other interneuron classes only briefly in the discussion, but yes, SOM interneurons are of course not the only candidates. We have extended the relevant discussion section as follows (l. 453):

Several cortical interneuron classes were ignored, including interneuron types that also inhibit the distal dendrite (Abs et al., 2018; Garcia-Clemente et al., 2017). In principle,

those interneurons could provide the suggested homeostatic control of dendritic activity equally well as SOM interneurons. We chose to refer to the dendrite-targeting interneurons as SOM interneurons, because those --- specifically deep layer Martinotti cells --- receive excitatory drive from the surrounding PC population (Murayama et al., 2009) and are therefore good candidates for the feedback inhibition modelled in Fig 3. Neurogliaform interneurons in layer 1 such as neuron-derived neurotrophic factor-expressing (NDNF) interneurons also target apical PC dendrites, but seem to receive primarily long-range, top-down inputs (Abs et al., 2018, Pardi et al., 2020).

5. In Fig. 3 it would be useful to also show the CVs for single spikes.

Thanks for this suggestion. However, we believe that Fig 3 is already quite information-dense, and adding the CV of interspike intervals of single spikes does not add much additional insight. It essentially looks like the CV distribution of the inter-event intervals, just slightly shifted to the right because of the occasional longer intervals that arise from the skipped bursts. Here's a direct comparison of all CV distributions for illustration:

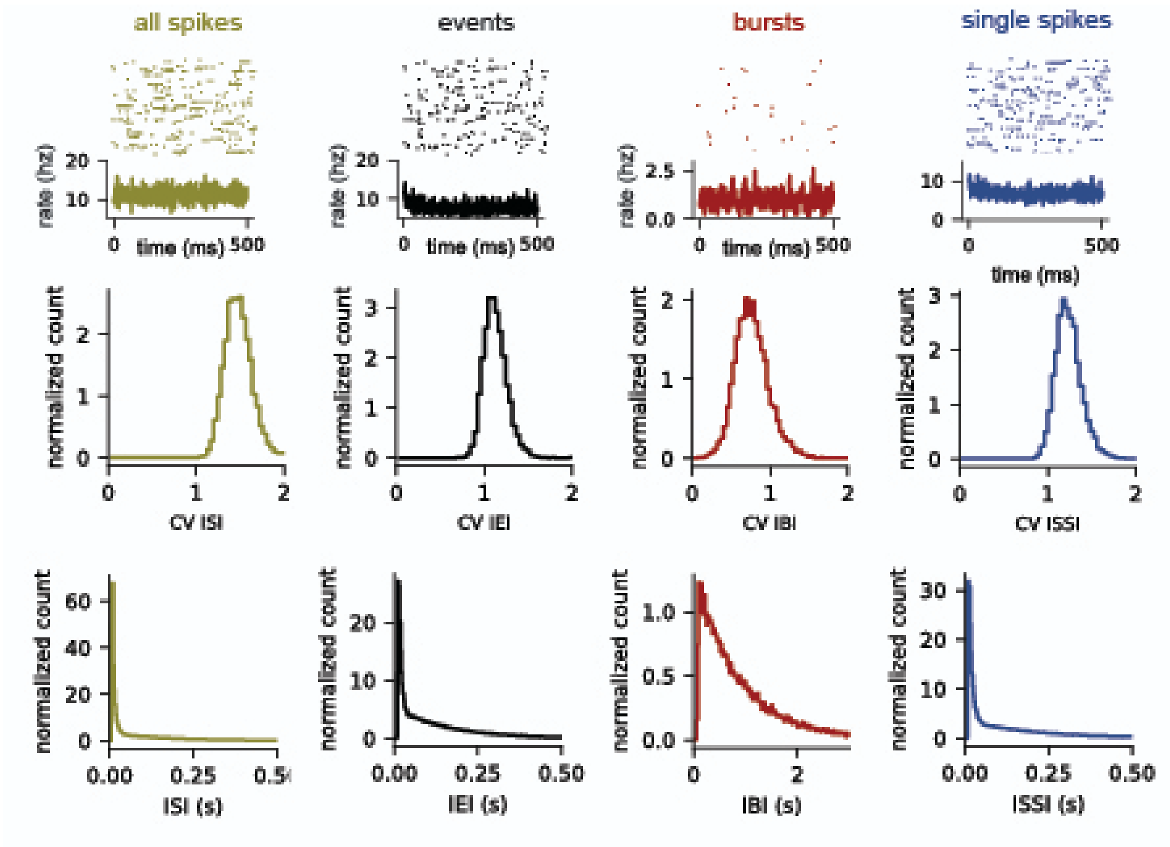

6. It should noted that Sacramento et al. introduced a learning rule for SOMs that is very similar to this one, but without explicit bursting mechanisms.

Although the rule introduced by Sacremento et al. (2017) is rate-based, we agree that it's a homeostatic inhibitory plasticity rule that is similar in nature and should be referenced in the discussion. We extended a suitable paragraph as follows (l. 378):

> *In our simulations, we used bursts as a proxy for dendritic activity of L5 pyramidal neurons, but dendritic membrane potential or currents (Fig S1; Urbanczik & Senn, 2014), or local chemical signals could be equally suitable. Earlier models of dendritic (inhibitory) plasticity also exploited local dendritic signals, e.g., to learn dendritic predictions of somatic inputs by comparing compartmental membrane potentials (Urbanczik & Senn, 2014; Sacramento et al., 2017).*

7. The use of ISP within PVs (Fig. 3) seems to be an interesting point that I haven't seen before. The need for this should be extended and it should be discussed as a potential prediction. In addition, why do the PVs receive external input but the SOMs don't?

We thank the reviewer for raising this point. Inhibitory plasticity among the PV interneurons is included merely for convenience. It controls the firing rate of PVs to 10 Hz without the need to tune the excitatory L5 PC to PV connection and/or the external excitatory input to the PVs. However, there is an interaction with the amount of external drive to the PVs, because the strength of the recurrent connections among the PVs depends on the relation of the total excitatory input and the target rate. The more input, the higher the recurrent weights. This point, and the question why we did not include external input to the SOMs, is now discussed in the methods, see reply to the first major comment of reviewer 1.

8. Fig 3E: You state that "Weak (strong) external input leads to small (large) input fluctuations on the net input current" which is ambiguous because we don't know if you are saying that you changed the external input current to the somatic and dendritic compartments independently or if both were changed. Also, what are the values for the strong and weak external inputs? Please clarify.

We agree, the formulation of the somatic and dendritic inputs was ambiguous. We modified the caption as follows:

> *Weak (I^s = 700 pA, I^d = 300 pA) or strong (I^s = 1500 pA, I^d = 500 pA) external input leads to small or large input fluctuations on the net input current, respectively.*

9. Fig 3F: Same Q as 3E, are you varying independently or together?

Thanks. The dendritic and somatic input vary together, and we have updated the caption accordingly:

> *(F) Standard deviation (left) and the mean (right) net input currents for soma (black) and dendrites (red), when increasing the external somatic (black X-axis) and dendritic (red X-axis) inputs simultaneously.*

10. Fig 3G: in the text, you state that “this internally generated noise has the effect of smoothing out the input-output function of the network, such that transient inputs to the network can be represented in a graded rather than an all-or-none fashion” but only show the smoothing for the bursting. Does this happen for the normal spiking as well? Please clarify.

Indeed, the firing rate response to dendritic inputs is of course also linearized, as is expected from an AI state, see below. However, because the purpose of this panel was to relate back to Fig 1, we hope it's ok if we do not go into detail on this point in the paper (once we start this, we'd have to provide 4 additional plots showing burst/firing rate responses to somatic/dendritic input). Instead, we limited the scope of the claim rather than broaden the discussion at this point (l. 285):

> *Functionally, this internally generated noise has the effect of smoothing out the input-output function of the dendrites, such that transient dendritic inputs are represented in the burst rate in a graded rather than an all-or-none fashion (Fig 3G, cf. Fig 1B).*

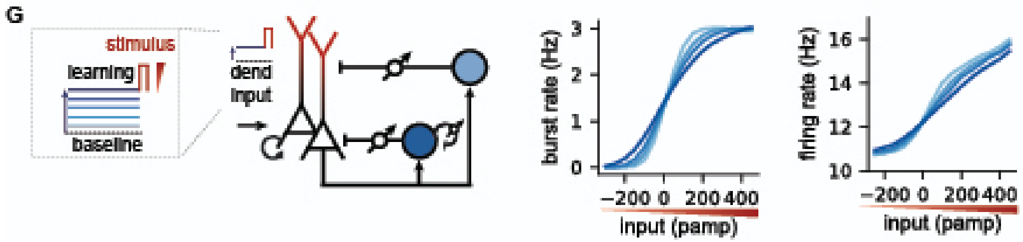

11. Fig 4B: Why are there two high somatic inputs chosen and one is a negative current? Is it a typo and there shouldn’t be two high somatic inputs: the -480pA input is probably the low dendritic input?

Thanks for reading so thoroughly. We corrected the text in the caption and also added the variability of the noisy background input:

> *Alternating and opposite pulse inputs are delivered to the somatic and dendritic compartment ($I^{s,high}$ = 800 pA, $I^{s,low}$ = 500 pA, $I^{d,high}$ = 105 pA, $I^{d,low}$ = - 480 pA, $\sigma^s = \sigma^d$ = 450 pA).*

It is correct that the mean value of the low dendritic current is negative (-480 pA). A noisy background input with negative mean, but high variance, can still drive burst activity.

12. There is something strange in Eq.17 you state that it should rescale the inputs to match that of the burst fraction (BF) or event rate but for example if the min value of the BF is 0 and the max is 1 then what you get out is just the same input which suggests it wasn't scaled at all.

This equation is now obsolete, because we now use a different way of comparing input and output (see response to your question 2 above).

13. Fig S3: Letters referring to the wrong parts of the figure: No competition should be B-C not A-B and competition should be D-E not C-D.

Thanks, corrected.
